# Application of Activated Carbon Banana Peel Coated with Al_2_O_3_-Chitosan for the Adsorptive Removal of Lead and Cadmium from Wastewater

**DOI:** 10.3390/ma15030860

**Published:** 2022-01-24

**Authors:** Denga Ramutshatsha-Makhwedzha, Richard Mbaya, Mapula Lucey Mavhungu

**Affiliations:** Department of Chemical, Metallurgical and Materials Engineering, Pretoria West Campus, Tshwane University of Technology, Private Bag X680, Pretoria 0183, South Africa; Mbayar@tut.ac.za (R.M.); MoropengL@TUT.ac.za (M.L.M.)

**Keywords:** adsorption, univariate optimization, wastewater, banana peel activated carbon, nanocomposite, toxic metals

## Abstract

This study was aimed at evaluating the adsorption capacity of novel banana peel activated carbon (BPAC) modified with Al_3_O_2_@chitosan for the removal of cadmium (Cd^2+^) and lead (Pb^2+^) from wastewater. Characterization techniques such as X-ray diffraction, scanning electron microscopy, transmission electron microscopy, Fourier transformed infrared (FTIR) spectroscopy, and Brunauer–Emmett–Teller analysis confirmed the synthesized BPAC@Al_3_O_2_@chitosan composite material. The univariate approach was used to study the influence of different experimental parameters (such as adsorbent mass, sample pH, and contact time) that affects simultaneous removal of Cd^2+^ and Pb^2+^ ions. Kinetic results showed that adsorption favored the pseudo-second-order kinetic model, whereas the adsorption of Cd^2+^ and Pb^2+^ was best described by the Langmuir model and the adsorption capacity for Cd^2+^ and Pb^2+^ was 46.9 mg g^−1^ and 57.1 mg g^−1^, respectively, for monolayer adsorption. It was shown the BPAC composite can be re-used until the third cycle of adsorption–desorption (% Re > 80). Based on the obtained results, it can be concluded that the prepared BPAC@Al_3_O_2_@chitosan composite material is cost effective, as it is generated from waste banana peels and can be re-used. In addition, the prepared material was able to remove Cd^2+^ and Pb^2+^ up to 99.9%.

## 1. Introduction

Toxic heavy metals are one of the main contaminants of water resources [1]. The industries (such as petroleum refining, textile dying, mining operations, and ceramics) are the point sources where metal pollutants migrate from, ending up in near and far regions and causing pollutions of surface-water resources [2].

Some of the toxic and carcinogenic heavy metals include cadmium (Cd) and lead (Pb). These metals also bear significant properties when they appear in a form of compounds such as lead tungstate (PbWO_4_) and cadmium tungstate (CdWO_4_). They are also a scintillating crystal that has been widely explored in physics, with better mechanical, chemical, and optical properties [3,4]. These properties make them suitable for various applications, which include high-energy physics, spectroscopy, and gamma detectors [5]. However, pure metal cadmium (Cd) and lead (Pb) are some of the most toxic and carcinogenic heavy metals. Their major sources are wastewater treatment plants from different process industries [6]. Cd and Pb are very poisonous metals and are known to have adverse impacts on the human body, including the central nervous system, kidneys, and liver [6]. Therefore, it is important to remove and minimize their adverse effects from industrial effluents by reducing them to an acceptable concentration.

With regard to the effective removal of metals from water, various techniques such as filtration [7], ion exchange [8], reverse osmosis [9], chemical precipitation [10] (Int, 2019), and adsorption [11] were previously explored. Amongst these techniques, adsorption is highly favored due to its potential to remove both organic and inorganic pollutants even at a trace level [12]. Adsorption is one of the most efficient methods for the removal of metals due to its high efficiency, easy operation, and low cost [13].

Adsorption performance is mainly dependent on the type of adsorbent used. Consequently, various studies were conducted on the development of efficient, cost-effective, and environmentally friendly adsorbents [14]. Adsorbents such as metal oxides [15], zeolites [16], metal-organic frameworks (MOFs) [17], and carbonaceous materials [18] have been prepared and used for the removal of metal ions from wastewater. Among carbonaceous materials, activated carbon (AC) has received enormous research interest because it is an economical and reliable adsorbent. This is due to its good characteristics, such as large surface area, highly porous structure, and high catalytic activity. These are valuable in the adsorption of different pollutants, including metals [19,20]. In addition, AC can be prepared from waste materials through chemical and physical processes. As a result, it contributes to making the process of production economical [21]. The chemical and physical processes pave the way to achieving AC with effective and desired adsorption properties [2]. Furthermore, the adsorption capacity of AC can be enhanced by modification and functionalization using metal oxides [19], polymers [22], and surfactants [23], amongst others. Metal oxides are widely used in the removal of contaminants because they are cheap and broadly manufactured; they also have high mechanical properties and resistance to thermal decomposition [24].

In this study, AC derived from waste banana peels modified with chitosan and Al_2_O_3_ nanoparticles (BPAC@Al_2_O_3_@chitosan) was used as an adsorbent for the removal of Pb^2+^ and Cd^2+^. Aluminum oxide (Al_2_O_3_) nanoparticles were chosen because they have a high surface area, are chemically stable, non-poisonous, easy to use, and also have many hydroxide groups, which makes them suitable adsorbents [24]. Nanoparticles have a mean diameter of 53 nm, and the modified diameter ranges from 68 to 87 nm [25]. Metal-oxide nanoparticles can be prepared using the following methods described in the literature: vapor decomposition [26], coprecipitation [27], combustion [28], and sol-gel [29]. The sol-gel method makes use of wet-chemistry reactions for metal oxide nanoparticle preparation, and it has the advantage of intimate molecular-level mixing of the components [30].

Nano metal oxides (NMO) have been reported to show good adsorption capacity and selectivity, thus making them a good adsorbent for metal removal [31,32]. Nevertheless, their small size contributes to the oxides having large surface energy, which may lead to poor stability [33]. Chitosan, a biopolymer, was chosen because it improves the stability state of NMOs. Hence, the impregnation of natural materials onto porous supports was conducted using a synthetic polymeric host such as chitosan. A polymeric sorbent such as chitosan was used for water decontamination because it is an eco-friendly biopolymer that has attractive properties, such as biodegradability, biocompatibility, non-toxicity, is widely used, and is a low-cost adsorbent for metal-ion removal because of its high ratio of hydroxyl to amine groups [34].

This study examines adsorption characteristics for Cd^2+^ and Pb^2+^ removal on modified waste banana peel activated carbon (BPAC) by Al_2_O_3_ nanoparticles and chitosan biopolymer. The physicochemical properties of both modified and unmodified BPAC were determined using X-ray diffraction (XRD), scanning electron microscopy (SEM), transmission electron microscopy (TEM), and Brunauer–Emmett–Teller (BET) surface analysis. Experimental parameters (such as pH, adsorbent mass, initial concentration, and contact time) affecting the removal of Cd^2+^ and Pb^2+^ were optimized using the univariant approach.

## 2. Materials and Methods

### 2.1. Materials and Reagent

Aluminum oxide (Al_2_O_3_), chitosan, oxalic acid (C_2_H_2_O_4_), and lead nitrate (Pb(NO_3_)_2_) were purchased from Sigma-Aldrich (St. Louis, MO, USA). Sodium nitrate (NaNO_3_) and cadmium sulphate (CdSO_4_∙8H_2_O) were obtained from Arkema (Johannesburg, South Africa). Nitric acid (HNO_3_) and sodium hydroxide (NaOH) were obtained from Merck Chemicals (Johannesburg, South Africa). Banana peels were collected from waste materials. The working solutions of Cd^2+^ and Pb^2+^ were prepared from a 1000 mg L^−1^ stock solution by making appropriate dilutions using distilled water. The pH of the solution was adjusted to the desired value using dilute solutions of HNO_3_ or NaOH.

### 2.2. Instrumentation

The morphologies, elemental compositions, and nanostructures of the synthesized materials were investigated using scanning electron microscopy, coupled with energy-dispersive x-ray spectroscopy (SEM, TESCAN VEGA 3 XMU, LMH instrument, Brno, Czech Republic) and transmission electron microscopy (TEM, JEM-2100, JEOL, Tokyo, Japan). The X-ray diffraction (XRD) spectra were recorded using a PANalyticalX’Pert X-ray Diffractometer. The Fourier transformed infrared (FTIR) spectroscopy of (BPAC@Al_2_O_3_@chitosan) was obtained on a Perkin Elmer Spectrum. The FTIR spectra of samples were obtained in the range of 500–4000 cm^−1^ and an average of 32 scans were taken. The Brunauer–Emmett–Teller (BET) specific surface area and the pore distributions of the materials were measured using an ASAP 2020 porosimeter TriStar II 3020 3.00 surface area and porosity analyzer (Micromeritics, Norcross, GA, USA). The inductively coupled plasma emission spectroscopy (ICP-ES) (ICPE-9000, Shimadzu, Kyoto, Japan) was used for the quantification of analytes in sample solutions.

### 2.3. Synthesis of Banana Peel Activated Carbon (BPAC)

The synthesis of activated waste banana peels was adopted from the literature with some modifications [35]. Waste banana peels were collected, washed, and dried in the oven at 100 °C. Dried peels were then crushed into a fine powder using a pestle and motor. The powder was calcined at 600 °C, forming a powder in the furnace for 1 h. The powder was thereafter activated using 1 M H_2_SO_4_ for 12 h.

### 2.4. Activation of the Banana Peel Powder

Chemical activation, unlike physical activation, allows pyrolysis and activation to occur in a single process [36]. A preference for using chemical activation over physical has been reported because of its porosity, large surface area, and the pore size achieved on the material after activation. Consequently, chemical activation was used as an alternative process in the production of activated carbon from waste material [36].

In this study, chemical activation was investigated using H_2_SO_4_ and KOH chemical agents at room temperature. The mass of 5 g adsorbent material was in contact with 250 mL of 1 M chemical agent and the solution was stirred for 12 h at room temperature. The material was washed several times using distilled water until the pH of the solution was neutral. Adsorbent recovered (activated carbon) was then filtered using filter paper and dried at 50 °C using an oven. This process was crucial, as it impregnates the raw carbon with a relevant functional group that assists it in absorbing heavy metals.

### 2.5. Preparation of BPAC@Al_2_O_3_@chitosan Nanocomposite

The BPAC@Al_2_O_3_@chitosan composite was prepared following a method reported in the literature, with some modifications [34]. Briefly, chitosan (0.25 g) was added into 0.2 M oxalic acid solution with continuous stirring at 50 °C to form a viscous gel. Thereafter, 0.75 g acid-treated AC together with 0.5 g of Al_2_O_3_ were added to the gel and stirred further at 50 °C for 12 h. After this step, the BPAC@Al_2_O_3_@chitosan composite (3:2:1) was produced by dropwise addition of gel to 0.7 M NaOH solution in a precipitation bath. The composite was left stirring overnight to mature and the bio-nano composite beads were separated from the NaOH solution by filtration and washed with deionized water until reaching a natural pH. The prepared beads were then dried in the vacuum oven. Adsorbents of ratio 2:1:1 and 3:1:2 were synthesized following the same procedure for comparison study.

### 2.6. Adsorption Experiments

The batch-wise adsorption of Pb^2+^ and Cd^2+^ on the BPAC@Al_2_O_3_@chitosan was carried out. The adsorption capacity of the BPAC@Al_2_O_3_@chitosan towards the target analytes was determined by introducing 20 mL of the pH-adjusted solution containing water samples of initial concentrations of 20–100 mg L^−1^ of Cd^2+^ and Pb^2+^ into 100 mL polypropylene bottles containing a mass of 0.005–0.6 g of the sorbent. The mixture of the sorbent material and target analyte solution was ultrasonicated at room temperature for 5–60 min. The sorbent was then separated from the solution and the solution was filtered before analysis on the ICP. The amount of Cd^2+^ and Pb^2+^ adsorbed onto the sorbent was calculated using the formula for adsorption capacity and adsorbed efficiency. The equation was well described and defined by Alasai et al. [37].

### 2.7. Isotherm Studies

To establish the relationship between the absorbed amount of Cd^2+^ and Pb^2+^ onto BPAC@Al_2_O_3_@chitosan together with its equilibrium concentration, Langmuir and Freundlich’s models were used. The linear model of Langmuir was reported by Naseem et al. [38]. The Langmuir constants that relate to adsorption capacity (mg g^−1^) are q_max_ and K_L_. The constants can be calculated from the slopes of the linear plots Ce/q_e_ versus C_e_, respectively. The value of the R^2^ coefficient shows how good the experimental data were on the model.

The Freundlich isotherm can be used for the non-ideal sorption that involves a heterogeneous surface energy system, as well as multilayer sorption [39]. The linear equation for Freundlich can be expressed as follows [38]. K_F_ is the Freundlich constant, which relates to adsorption capacity, and *n* is the Freundlich exponent. The Freundlich constant and exponent are determined from the intercept and slope of the linear plot of ln q_e_ versus ln C_e_.

### 2.8. Adsorption Kinetics

The optimum time required for the adsorption of Cd^2+^ and Pb^2+^ to achieve equilibrium was determined at a function of time in the range of 5–90 min of 20 mg L^−1^ at a pH of 6 at room temperature. To examine the adsorption behavior, pseudo-first order, pseudo-second order, and intra-particle diffusion models were used to analyze the obtained experimental data [37].

q_t_ is the adsorption capacity at time t (mg g^−1^), and k_1_ and k_2_ are the rates constant (min ^−1^). The first-order rate constant can be calculated from the slope and the intercept of the plot [11]. The second-order constant k_2_ can be determined experimentally from the slope and intercept of the plot of t/q_t_ versus t, one of the most important factors to be considered in the adsorption process is the prediction of the rate-limiting step [40]. This is ruled by the adsorption mechanism, which is essential for the design mechanism. The best-known technique to pinpoint this mechanism is by fitting the intra-particle diffusion plot. The intraparticle diffusion equation is as follows [33].

### 2.9. Application in Real Wastewater

Wastewater samples were collected from the Pretoria wastewater treatment plant (Gauteng Province, South Africa). A wastewater sample in a 2000 mL polyethylene bottle was stored in the refrigerator at a temperature of 4–8 °C after sampling. The polyethylene bottle used was initially washed and soaked in 1% nitric acid and rinsed with distilled water three times. This was done to avoid possible contamination.

### 2.10. Reusability of BPAC@Al_2_O_3_@chitosan Composite

The reusability studies of the BPAC@Al_2_O_3_@chitosan adsorbent were investigated using the adsorption/desorption cycles. This was done using the optimum experimental conditions (pH 6, 0.1 g, 30 min) obtained during optimization. Desorption of the analytes from the adsorbent was done by washing the adsorbent with 3 M nitric acid. In addition, deionized water was used to wash the adsorbent before drying in the oven and reuse. The cycle of the adsorption–desorption process was repeated until the adsorbent was exhausted.

## 3. Results

### 3.1. Preliminary Studies

The preliminary studies were conducted to determine the most suitable activation agent between H_2_SO_4_ and KOH. The BET surface areas of the pristine BPAC-H_2_SO_4_ and BPAC-KOH were studied and the results are shown in Table 1. Results show that high surface area and pore volume were obtained with activated carbon by H_2_SO_4_. The low pore volume of BPAC calcined at 600 °C could have been due to the closed pore that resulted in the material being less porous. The carbon activated by 1 M of H_2_SO_4_ exhibited a large surface area of 361.86 (m^2^ g^−1^). Results show that chemical activation on BPAC by H_2_SO_4_ enhanced the surface area of AC, giving it suitable properties that are favorable in the adsorption of heavy metals. Hence, 1 M of H_2_SO_4_ was the method chosen for activation of carbon in this study going forward.

### 3.2. Physicochemical Properties of the Adsorbents

**Figure 1:**
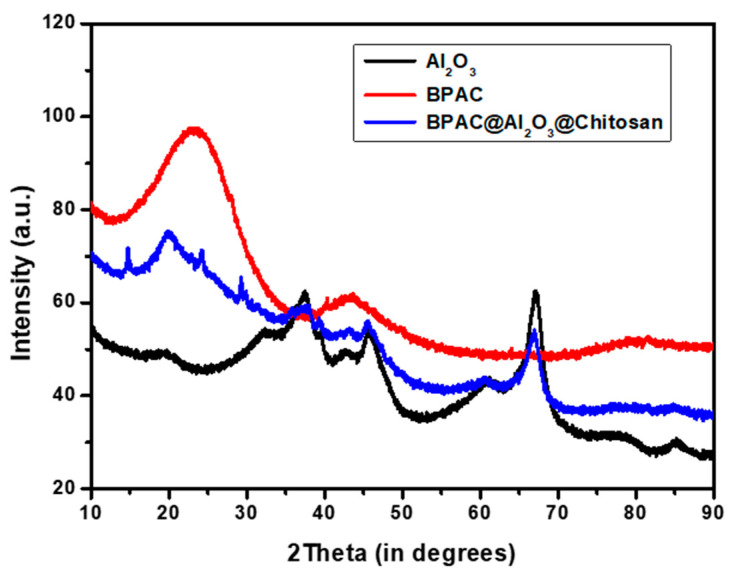
XRD spectra of BPAC, Al_2_O_3_, and BPAC@Al_2_O_3_@chitosan composite.

Figure 1 shows the XRD patterns of BPAC, Al_2_O_3_, and BPAC@Al_2_O_3_@chitosan. X-ray diffraction patterns were analyzed by scanning from the 5.00–90.00° 2-theta range. The BPAC pattern showed an amorphous structure that also appeared on the composite material at 2-theta 5–30°. The broad peaks that were observed in Al_2_O_3_ disappeared during the incorporation of chitosan; hence, the structure of the BPAC@Al_2_O_3_@chitosan material was partial crystalline with broader peaks from 2-theta 35–40° and 42–48°. The XRD pattern of BPAC@Al_2_O_3_@chitosan indicates the presence of three distinct Al_2_O_3_ peaks at 2-theta values of 36.0°, 45.5°, and 67.0°. Al_2_O_3_ nanoparticles have many properties that will assist in the adsorption of metals from wastewater.

**Figure 2:**
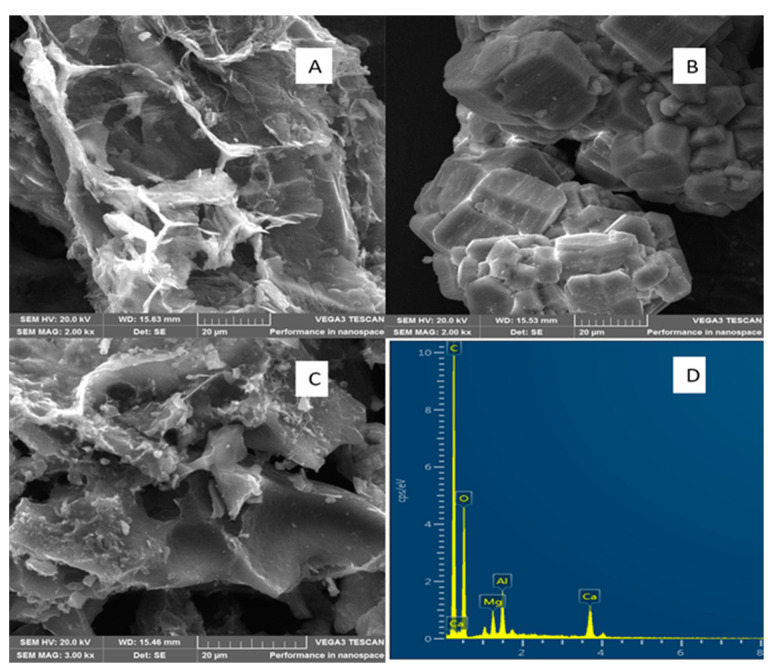
SEM image of (**A**) BPAC, (**B**) Al_2_O_3_, (**C**) BPAC@Al_2_O_3_@chitosan, and (**D**) SEM-EDX spectra of BPAC@Al_2_O_3_@chitosan. SEM HV = 20 kv.

Figure 2 shows the SEM morphological structure of BPAC, Al_2_O_3_, and composite materials. The SEM image of BPAC in Figure 2A showed an interconnected structure of BPAC, with large openings on the surface of the material that resembled the formation of pores. The SEM image of Al_2_O_3_ in Figure 2B showed irregular shapes that were agglomerated together, which include a combination of various shapes such as cubic, hexagonal, and spherical, amongst others. The irregular structure of Al_2_O_3_ makes it easy for the adsorption of metal ions to take place at different parts of the adsorbent. On the other hand, the SEM image of the BPAC@Al_2_O_3_@chitosan composite in Figure 2C still resembled the pore structures even after the coating of BPAC with chitosan and the incorporation of Al_2_O_3_ nanoparticles [2]. The elemental composition confirms the successful preparation of the BPAC@Al_2_O_3_@chitosan composite (Figure 2D).

**Figure 3:**
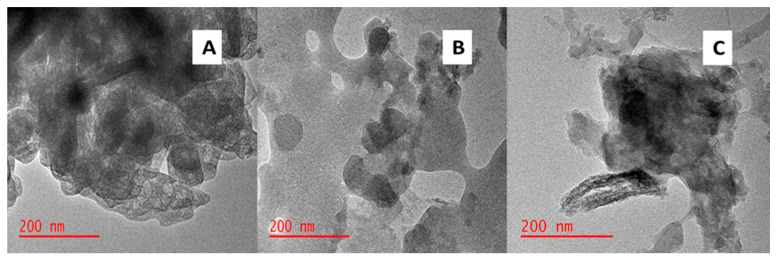
TEM images of (**A**) BPAC, (**B**) Al_2_O_3_, and (**C**) BPAC@Al_2_O_3_@chitosan.

The TEM technique was used to examine the microstructure of BPAC, Al_2_O_3_, and BPAC@Al_2_O_3_@chitosan composite materials. The TEM image displayed in Figure 3A shows the morphology of BPAC with binding micro-structures, as confirmed by the SEM image (Figure 2A). Figure 3B attached shows the image that resembled dispersed particles with various shapes, including hexagonal and spherical, whereas the darker side of the image in Figure 3C resembled the Al_2_O_3_ nanoparticles [34]. Results further show that chitosan was homogenously functionalized on AC with no aggregation formed, which means that the chitosan was highly dispersed in aqueous media [41]. The BET surface area of BPAC@Al_2_O_3_@chitosan was found to be 140.4 m^2^ g^−1^. The high surface area obtained is preferred for the adsorption process because it provides more active sites for the reaction to take place.

**Figure 4:**
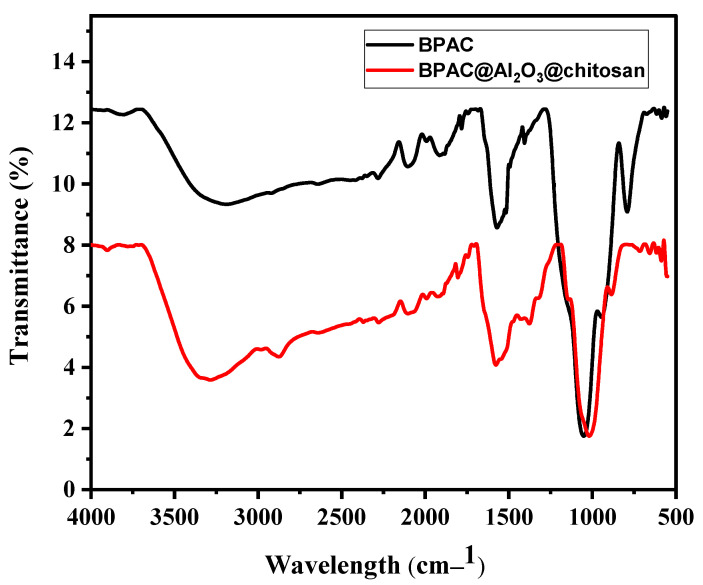
FTIR spectra of BPAC and BPAC@Al_2_O_3_@chitosan.

Figure 4 shows the FTIR spectroscopy of BPAC and BPAC@Al_2_O_3_@chitosan, which was used to determine the functional groups present. The spectrum confirmed that the BPAC and BPAC@Al_2_O_3_@chitosan had almost similar infrared spectra bands between the regions of 1022 and 3500 cm^−1^. The structures of carboxylic acid, alcohol, and phenols from the existence of the hydroxyl (OH) group were indicated by the broad peak at 3280 cmÀ1. The bands at 1570 cmÀ1 and 1387 cmÀ1 were assigned to C=O stretching vibrations of the carboxyl and carbonyl groups and C=C stretching vibrations, respectively. The peaks at 2869 cmÀ1 could be assigned to aliphatic, asymmetrical, and symmetrical C–H stretching, whereas the presence of C–O stretching vibrations could be assigned to the weak peak at 1022 cmÀ1. The hydroxylic group that was observed in both BPAC and BPAC@Al_2_O_3_@chitosan is important for the adsorption of metals [42].

### 3.3. Adsorption Studies

#### Selection of Adsorbent

**Figure 5:**
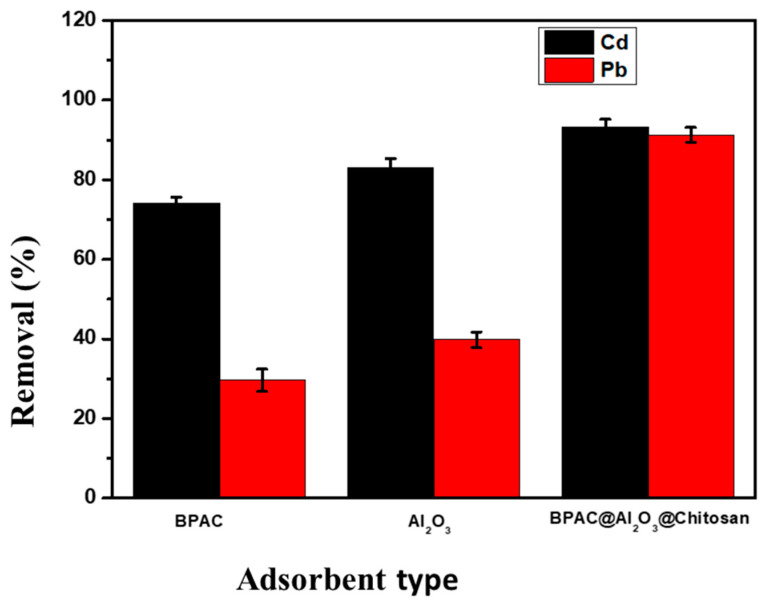
Adsorption of Cd^2+^ and Pb^2+^ on BPAC, Al_2_O_3_, and BPAC@Al_2_O_3_@chitosan composite material at a contact time of 30 min, adsorbent mass of 0.1 g, and initial concentration of 20 mg L^−1^.

Preliminary studies were conducted to determine which of the synthesized adsorbent had a better affinity towards the target metal ions and the analytical response, displayed in terms of %R in Figure 5. Results show that BPAC@Al_2_O_3_@chitosan was the best adsorbent for the removal of Cd^2+^ and Pb^2+^, with 94% removal. This is likely due to the incorporation of both chitosan and Al_2_O_3_ on BPAC. Hence, further experiments were conducted using the BPAC@Al_2_O_3_@chitosan adsorbent.

### 3.4. Optimization of the Adsorption Batch Method

#### 3.4.1. Effect of pH

**Figure 6:**
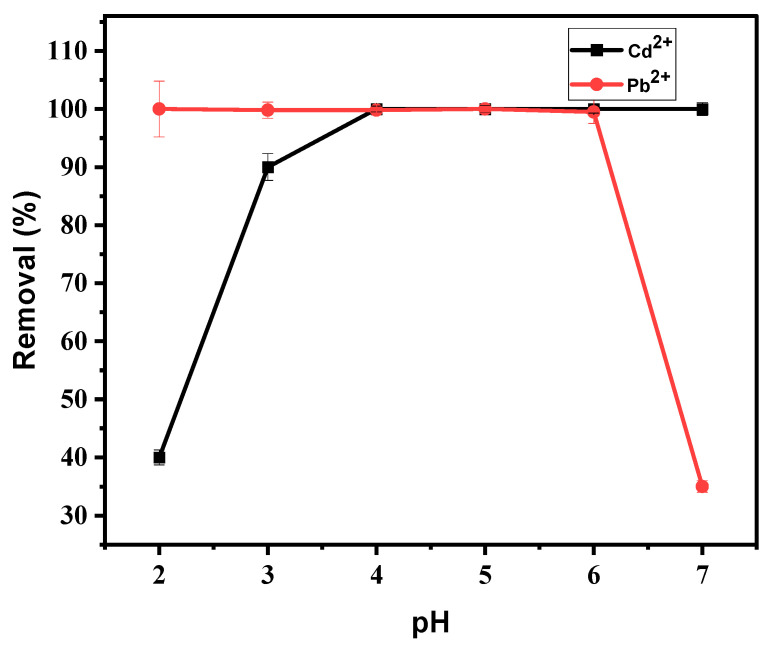
Effect of pH on the adsorption of Cd^2+^ and Pb^2+^ using BPAC@Al_2_O_3_@chitosan material at a duration of 30 min, the adsorbent mass of 0.1 g, and initial concentration of 20 mg L^−1^.

The effect of pH is one of the important parameters in the adsorption of metal ions. This is due to the surface-charge density of the adsorbent together with the metallic speciation, which depends on pH [43]. Adsorption of Cd^2+^ and Pb^2+^ was evaluated in the pH range from 2 to 7, as shown in Figure 6. A pH of more than 7 was avoided, as lead precipitates beyond pH 6 [44]. The rest of the parameters, which included adsorbent mass, contact time, and initial concentrations, were kept constant at 0.1 g, 30 min, and 20 mg L^−1^, respectively. Figure 6 shows that the percentage removal of Cd^2+^ increased with an increase in pH from 2 to 4; when it reached pH of 4, the trend became constant at 98% removal until pH 6. On the other hand, at a pH of 2–6, the removal of Pb^2+^ ranged from 98–100%, and when it reached a pH of 7, the percentage removal dropped significantly. The absorption was quite weak at the lower pH. In such an environment, the surface of the adsorbent became more protonated and the access of positive metal ions was hindered as a result of the repulsive force. There was also competitive adsorption between H^+^ and metal cations at the negative active centers on the surface of the adsorbent. Increasing the pH increased the negative charge on the surface of the adsorbent and a significant improvement in adsorption was found [45]. The binding mechanism was influenced by the electrostatic attraction between the external surface of BPAC@Al_2_O_3_@chitosan and Cd^2+^ and Pb^2+^ ions. At pH 7, a decrease in the adsorption of Pb^2+^ ions were due to the formation of soluble hydroxylated complexes and their competition with active centers. Similar results were also reported by Vijayalakshmi et al. [44] on the effect of pH on Pb^2+^ removal. Therefore, the optimum pH that was chosen for both metals was pH 6, as it was within the range of the optimum removal.

#### 3.4.2. Effect of Adsorbent Mass

**Figure 7:**
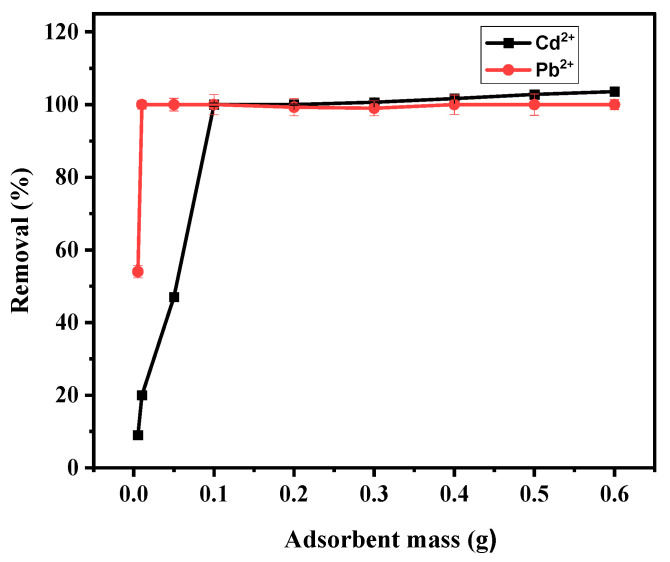
Effect of adsorbent mass on the adsorption of Cd^2+^ and Pb^2+^ using BPAC@Al_2_O_3_@chitosan material at a duration of 30 min, pH of 6, and initial concentration of 20 mg L^−1^.

Another important parameter in the adsorption of metals is adsorbent mass (AM). Hence, the effect of BPAC@Al_2_O_3_@chitosan composite mass on the removal of Cd^2+^ and Pb^2+^ was studied in the range of 0.005–0.6 g. Results shown in Figure 7 indicate that an increase in adsorption of Cd^2+^ was observed when adsorbent mass increased from 0.005 g to 0.1 g, and when it reached 0.1 g the trend became constant (Figure 7). The sharp increase was due to the accessibility of more active sites because of an increase in adsorbent dosage [46]. Pb^2+^ had a sharp increase from 0.005 and maintained the constant trend that showed 100% removal starting from 0.01 g. The optimum AM was 0.1 g.

#### 3.4.3. Effect of Contact Time

**Figure 8:**
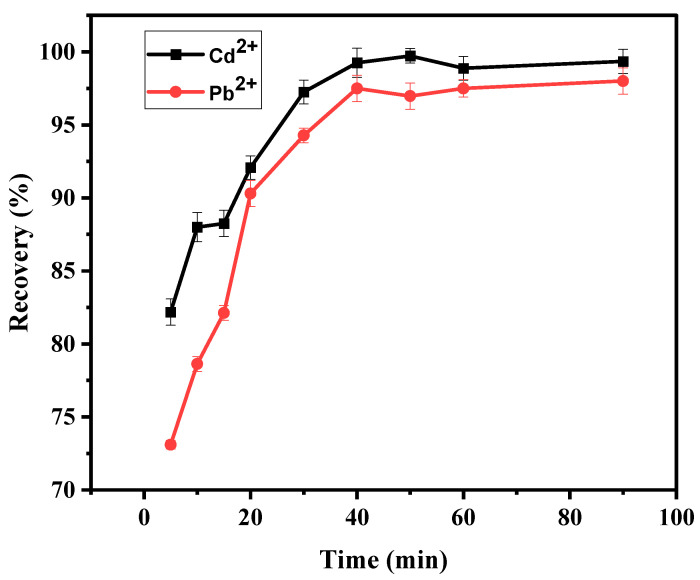
Effect of contact time on the adsorption of Cd^2+^ and Pb^2+^ using BPAC@Al_2_O_3_@chitosan material at an AM of 0.1 g, pH of 6, and initial concentration of 20 mg L^−1^.

Figure 8 shows adsorption for Cd^2+^ and Pb^2+^ ion uptake versus contact time at an initial concentration of 20 mg L^−1^. The effect of contact time played an important role in evaluating the competence of the material in adsorption. Results show that Cd^2+^ increased gradually from 82 to 99% when the time increased from 5 to 40 min, and equilibrium was reached at 40 min. The Pb^2+^ removal showed a sharp increase from 73 to 91% at 5 min to 20 min. It then increased gradually from 91 to 97% at 20–40 min before it reached equilibrium. Results show that the percentage recovery of Cd^2+^ and Pb^2+^ happened at a higher rate in the initial contact time due to the empty active sites on the BPAC@Al_2_O_3_@chitosan composite surface. It is eminent that at initial contact times the gradient of initial metal concentration is high in the solution, and as time increases the gradient decreases [46]. The highest adsorption% recovery of Cd^2+^ and Pb^2+^ at 40 min was determined to be 99 and 97%, respectively. Therefore, the optimum time is 40 min for adsorption of the studied analytes.

## 4. Discussion

### 4.1. Sorption Isotherms

**Figure 9:**
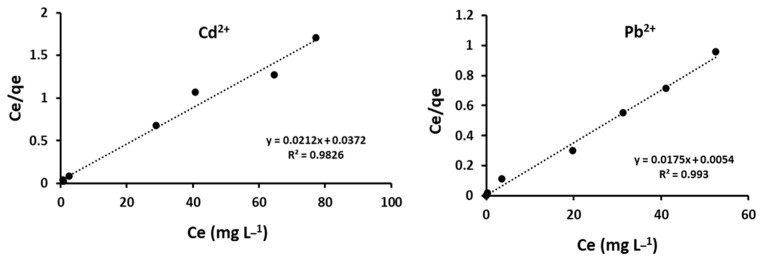
Langmuir isotherm model for Cd^2+^ and Pb^2+^ using BPAC@Al_2_O_3_@chitosan.

Adsorption isotherms provide a detailed study on the relationship between the adsorption of metal ions into the adsorbent material [38]. The isotherm experiments were performed using the optimum parameters at 298 K and initial concentrations ranging from 5 to 100 mg L^−1^. Figure 9 shows the representative graphs for the Langmuir isotherm equations. The values for the Langmuir regression coefficient (R^2^) were found to be 0.983 and 0.993 for Cd^2+^ and Pb^2+^, respectively. Results show that R^2^ values for both Cd^2+^ and Pb^2+^ were close to unity, which shows that adsorption was best interpreted by the Langmuir model. This indicates that the uptake took place on the homogenous surface by monolayer sorption [47].

The Langmuir constant (K_L_) was also used in the prediction of the affinity between the Cd^2+^ and Pb^2+^ cations and the adsorbent [37]. The low values of K_L_ show that BPAC@Al_2_O_3_@chitosan had a high affinity towards Cd^2+^ and Pb^2+^ (Table 2). The Langmuir isotherm can be expressed in terms of the dimensionless constant factor (R_L_), which shows important characteristics of the model [11]. The R_L_ results give information on the favorability of the adsorption process [48]. The value of K_L_ shows the extent of the adsorption of metal ions towards the adsorbent. When K_L_ is 0, R_L_ becomes unity, and the adsorbed metal per gram of adsorbent (q_e_) increases with the concentration of the analytes in equilibrium (C_e_) (linear). In a case where K_L_ is very high, it shows strong adsorption and R_L_ becomes zero, which is an irreversible reaction. The calculated values of R_L_ were 0.01 and 0.03 for Cd^2+^ and Pb^2+^. This suggests that the prepared BPAC@Al_2_O_3_@chitosan composite was highly favorable to the adsorption of Cd^2+^ and Pb^2+^ ions from wastewater samples.

The Freundlich model is used to give information on heterogeneous surface energy and the adsorbent surface roughness. The Freundlich model also assumes that adsorption sites are distributed exponentially according to the heat of adsorption. The stronger adsorption sites become saturated first, and adsorption strength decreases when site occupation increases. The value of the Freundlich exponent (*n*) ranges from 1 to 10 for a normal binding condition [49]. The calculated values of *n* were found to be 7.59 and 1.60 for Cd^2+^ and Pb^2+^, respectively. The *n*-values show favorable conditions of the adsorbed metal ions. The maximum adsorption capacity of Cd^2+^ and Pb^2+^ was found to be 46.9 and 57.1.0 mg g^−1^, respectively.

### 4.2. Adsorption Kinetics

The mechanism of adsorption together with its potential rate-determining steps was investigated using kinetic models [50]. Results of the pseudo-first order, pseudo-second order, and intraparticle diffusion are reported in Table 3. Pseudo-first order is based on solid capacity, whereas a pseudo-second order model is applied effectively to the adsorption of analytes from an aqueous solution where there is chemisorption that involves valency forces and the sharing or exchanging of electrons between adsorbent and adsorbate [51].

**Figure 10:**
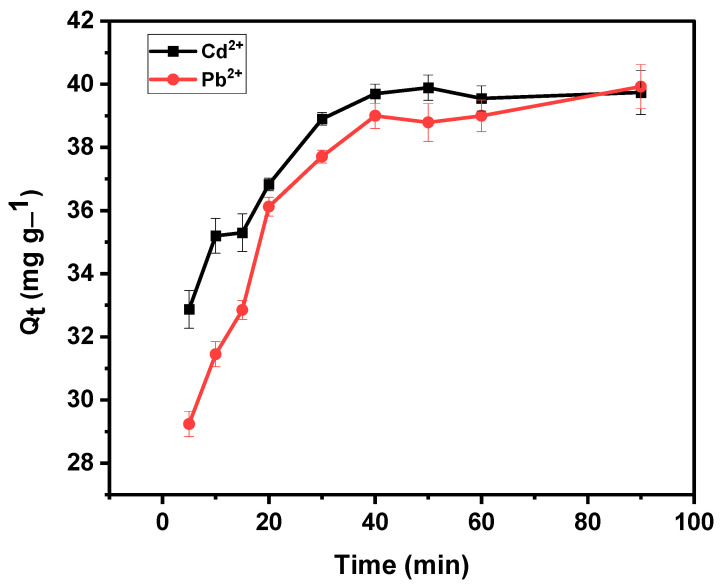
Adsorption capacity vs. time (min) on the adsorption of Cd^2+^ and Pb^2+^ using BPAC@Al_2_O_3_@chitosan material at an adsorbent mass of 0.1 g, pH of 6, and initial concentration of 20 mg L^−1^.

**Figure 11:**
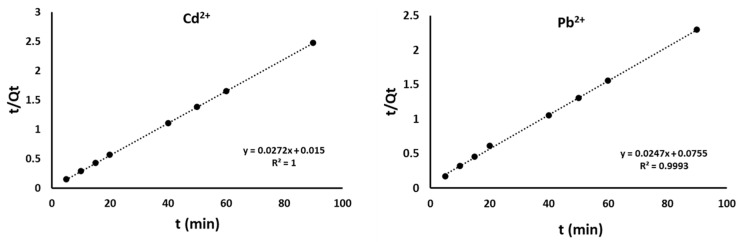
Pseudo-second-order equations for Cd^2+^ and Pb^2+^ using BPAC@Al_2_O_3_@chitosan.

The correlation coefficient (R^2^) was used to judge the suitability of the model as well as the agreement between the experimental and calculated data for adsorption capacity at time t (Q_t_) (Figure 10). The pseudo-second order equation gave the best fit with an R^2^ coefficient of greater than 0.999 on the sorption of both Cd^2+^ and Pb^2+^. Results show that a pseudo-second order binding mechanism is prevalent and the adsorption rate of Cd^2+^ and Pb^2+^ on BPAC@Al_2_O_3_@chitosan composite is determined by the chemisorption step, which involves the valence forces through the exchange of electrons between metal ions and adsorbent [49]. The rate constant (k_2_) and adsorption capacity (q_e_) from pseudo-second order kinetics were used to calculate the initial sorption rate (h), as well as t_1/2_, and the results are reported in Table 3. The time required to remove 50% of the targeted analytes at equilibrium is referred to as half-adsorption time (t_1/2_) [50]. Based on the obtained results, there was a high affinity between the adsorbent and the analytes with a short adsorption time of 1.2–2.7 min. The representative graphs for pseudo-second order equations are shown in Figure 11.

Based on the intra-particle model, the plot of Qt vs. t _½_ is linear if intra-particle diffusion is the only rate-limiting step. In this study, the plot did not pass through the origin, which implies that intra-particle diffusion was not the sole rate-limiting step. These show that there were other processes controlling the adsorption process at that particular time [43].

### 4.3. Application of Real Water Samples

To evaluate the applicability of the optimized method, wastewater was collected from the Pretoria treatment plant. The synthesized materials were applied to the removal of Cd^2+^ and Pb^2+^ that was found in wastewater. Results obtained from ICP-ES show that the wastewater had the following initial concentrations: 2.5 and 8.58 mg L^−1^ Cd^2+^ and Pb^2+^ on the influent, and 2.3 and 4.1 mg L^−1^ Cd^2+^ and Pb^2+^ on the effluent. Table 4 shows that the prepared BPAC @Al_2_O_3_@chitosan composite was able to remove Cd^2+^ and Pb^2+^ (%Re 92–99).

Table 5 shows a comparison study that was performed in wastewater using various adsorbent materials on the removal of metals [45,52,53,54,55,56,57]. Results show that the BPAC@Al_2_O_3_@chitosan composite was comparable with the literature and is amongst the best performing AC adsorbents made from agricultural precursors for the removal of Cd^2+^ and Pb^2+^ from wastewater.

### 4.4. Adsorption–Desorption Studies

**Figure 12:**
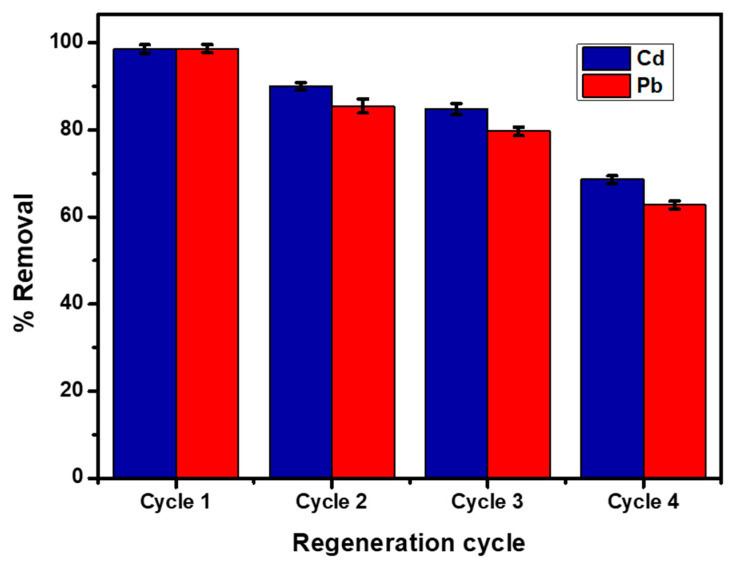
Regeneration for adsorption–desorption studies.

Regeneration study is important for an effective and economical adsorbent. To estimate the reusability of BPAC@Al_2_O_3_@chitosan adsorbent material, adsorption–desorption studies of Cd^2+^ and Pb^2+^ were performed four times with 3 M nitric acid. The adsorption percentage removal of BPAC@Al_2_O_3_@chitosan composite for the adsorption–desorption cycle is reported in Figure 12. Results show that the BPAC@Al_2_O_3_@chitosan composite was able to adsorb Cd^2+^ and Pb^2+^ for the third cycle with >80% regeneration efficiency. BPAC@Al_2_O_3_@chitosan composite adsorbent is very economical because it is made out of waste banana peels. No extra cost is needed when using waste banana peels as an adsorbent for wastewater treatment.

## 5. Conclusions

Banana peels are organic waste that can be used as an effective low-cost adsorbent for the removal of Cd^2+^ and Pb^2+^ from wastewater. They need to be utilized and managed well because they can create environmental pollution. Consequently, they may be used as an adsorbent for water treatment. Banana peels were successfully used as the starting material in the synthesis of BPAC@Al_2_O_3_@chitosan composite adsorbent. This study investigated the sorption of Cd^2+^ and Pb^2+^ metals by BPAC@Al_2_O_3_@chitosan composite material. Results show that pH is one of the parameters that are influential on the biosorption of Cd^2+^ and Pb^2+^ metal ions from aqueous solutions. The isotherms of the sorption of these metals were best described by the Langmuir isotherm model. The mechanism involved is considered to be an electrostatic attraction between BPAC@Al_2_O_3_@chitosan and Cd^2+^ together with Pb^2+^. The kinetics study shows that the data were best fitted by the pseudo-second-order model, which indicates that the chemisorption process and intra-particle diffusion were not the rate-limiting step. It can be concluded that the BPAC@Al_2_O_3_@chitosan has the ability to treat wastewater contaminated with Cd^2+^ and Pb^2+^.

## Figures and Tables

**Table 1:** Preliminary results for the activation of BPAC.

Activation	Surface Area (m^2^ g^−1^)	Pore Volume (cm^3^ g^−1^)	Pore Size (nm)
BPAC was calcined at 600 °C	2.275	0.155	256.0
BPAC-H_2_SO_4_	361.86	0.2294	25.36
BPAC-KOH	283.92	0.2245	31.63

**Table 2:** Adsorption isotherm parameters for the Langmuir and Freundlich models.

**Langmuir Parameters**					
**Cations**	**Q_max ex_**	**q_max_ (mg g^−1^)**	**K_L_ (L mg^−1^)**	**R_L_**	**R^2^**
Cd^2+^	43.0	46.9	0.57	0.03	0.983
Pb^2+^	57	57.1	1.75	0.01	0.993
**Freundlich Parameters**					
		**K_F_**	***n***	**R^2^**	
Cd^2+^		26.61	7.59	0.226	
Pb^2+^		14.67	1.60	0.684	

**Table 3:** Kinetics parameters for pseudo-first order, pseudo-second-order, and intra-particle diffusion.

		Cd^2+^	Pb^2+^
	qt exp	38	39.0
Pseudo-First order	k_1_ (min^−1^)	0.22	0.085
	q_e_ (mg g^−1^)	11.2 × 10^28^	1.0 × 10^28^
	R^2^	0.7155	0.886
Pseudo-Second order	k_2_ (g mg^−1^ min^−1^)	0.22	0.01
	qt (mg g^−1^)	40.65	41
	R^2^	0.999	0.999
	h (mg g^−1^ min^−1^)	33.05	15.3
	t_1/2_ (min)	1.2	2.7
Intraparticle Diffusion	k_id1_(g mg^−1^ min^−1^)	11.5	1.524
	C (mg g^−1^)	28.46	27.56
	R^2^	0.966	0.847

**Table 4:** Application of BPAC@Al_2_O_3_@chitosan composite material on the removal of Cd^2+^ and Pb^2+^ from real water samples.

Analytes	Influent Conc. (mg L^−1^)	%Re	Effluent Conc. (mg L^−1^)	%Re
Cd^2+^	2.5	99.8	2.3	99.3
Pb^2+^	8.58	97.4	4.1	92.8

**Table 5:** Summary of adsorption of heavy metals using activated carbon from the agricultural precursors.

Analytes	Adsorbents	Removal Efficiency (%)	Ref.
Pb (II)	Pistachio wood	99	[52]
Cu (II)	Magnetic activated carbon prepared from pistachio shells	94.5	[53]
Cr (VI)	Date press cake	>90	[54]
Cu^2+^, Ni^2+^, Pb^2+^	Sugarcane bagasse derived ZnCl_2_	66.4, 90, 99.9	[55]
Cu^2+^, Ni^2+^, Pb^2+^	KOH-activated carbon from banana peel	98.8, 99.2, 100	[56]
Cd (II)	Olive stones	95.3	[57]
Cd^2+^, Pb^2+^	Cherry pits	92.4, 94.5	[45]
Pb^2+^, Cd^2+^	BPAC @Al_2_O_3_@chitosan	<99 <92	This Work

## Data Availability

Data supporting the reported findings in this study are available upon request.
